# Health Care Use Among Cancer Patients With Diabetes, National Health and Nutrition Examination Survey, 2017–2020

**DOI:** 10.5888/pcd21.240066

**Published:** 2024-08-08

**Authors:** Ara Jo, Sarina Parikh, Nathalie Sawczuk, Kea Turner, Young-Rock Hong

**Affiliations:** 1 Department of Health Services Research, Management and Policy, University of Florida, Gainesville; 2 College of Public Health and Health Professions, University of Florida, Gainesville; 3 Now with School of Dental Medicine, University of Pennsylvania, Philadelphia; 4 Department of Health Outcomes and Behavior, Moffitt Cancer Center, Tampa, Florida; 5 Department of Gastrointestinal Oncology, Moffitt Cancer Center, Tampa, Florida

## Abstract

**Introduction:**

Diabetes is a common comorbidity among people with cancer. The objective of our study was to examine patterns of health care use among patients with cancer and either type 2 diabetes or prediabetes.

**Methods:**

We used data from the National Health and Nutrition Examination Survey (NHANES) for 2017–2020. The study population included US adults aged 18 years or older who were diagnosed with any cancer and type 2 diabetes or prediabetes (established by self-report and/or hemoglobin A_1c_ measurement). We used Poisson and multivariate logistic regression models to determine the effect of comorbidity on health care use, defined as health care visits and overnight stays in a hospital.

**Results:**

Of 905 cancer patients representing 27,180,715 people in the US, 24.4% had a type 2 diabetes diagnosis, and 25.8% had a prediabetes diagnosis. Patients with cancer and prediabetes had a significantly higher rate of health care visits (incidence rate ratio = 1.11; 95% CI, 1.01–1.22; *P* = .03) than patients with cancer only. We found no significant association between having cancer and type 2 diabetes and the number of health care visits or overnight hospital stays compared with patients with cancer only.

**Conclusion:**

More emphasis should be placed on optimal care coordination among people with cancer and other conditions, such as diabetes and prediabetes, to reduce the impact of comorbidity on health care use. Interventions integrated with technology to provide timely access to education on preventing or managing diabetes and prediabetes among cancer patients are warranted.

**Summary**

**What is already known on this topic?**

Cancer patients with multiple chronic diseases have unplanned hospitalizations because of a lack of appropriate care management. Multiple chronic diseases among people with cancer are associated with worse clinical outcomes and survivorship than among people with cancer only.

**What is added by this report?**

Patients with cancer and prediabetes had higher levels of health care use than patients with cancer only. A diagnosis of type 2 diabetes did not significantly affect health care use among patients with cancer.

**What are the implications for public health practice?**

Optimal care coordination and early management of prediabetes among patients with cancer via primary care may contribute to improving cancer survivorship.

## Introduction

Diabetes is a common comorbidity among people with cancer. As patients with cancer live longer due to advances in cancer treatment, rates of chronic conditions, such as diabetes, are expected to rise among people with cancer. People with type 2 diabetes (hereinafter, diabetes) have a substantially higher risk of cancer incidence and death, leading to poorer survivorship compared with people without diabetes (1,2). For example, people with diabetes, compared with people who do not have diabetes, have double the risk for liver and pancreatic cancers and have a higher risk of developing bladder, colon, and breast cancers (3). In addition, as cancer incidence and death rates have risen consistently over time, the comorbidity of cancer with other chronic diseases has gained attention (4,5). Despite these clinical outcomes, the research is limited on care delivery for people with cancer and other comorbidities.

People with cancer and comorbidities, compared with those who have cancer and no comorbidities, have greater unplanned use of health care services, including higher rates of unplanned hospital readmissions (6,7) and revisits to the emergency department (8). One study showed that among people with cancer and comorbidities, diabetes was the top reason for emergency department revisits (24% of all revisit encounters) (8). Another study found that the average length of hospital stay among people with cancer and diabetes was significantly longer than among patients with no comorbidity (9). In that study, the average length of a hospital stay among patients with colorectal cancer and diabetes who underwent surgery was almost 17 days, which is 3 days longer than among patients with cancer only (9). Furthermore, health care costs are of critical concern. A national study, which used 5 years of data from the Medical Expenditure Panel Survey (2010–2014), found that cancer patients spent on average 4 times more in annual health expenditures than noncancer patients (10). Early initiation of chronic disease prevention and management with a primary care physician can mitigate this financial burden.

Many patients with cancer face the challenges of comanaging cancer and chronic diseases. In a qualitative study conducted in 2021 and 2022 at 3 New York City hospitals among 15 women with breast cancer and either diabetes or prediabetes, participants reported a lack of information and education on managing chronic diseases and the burden of co-management with different providers (11). In addition, patients tended to prioritize cancer treatment over diabetes management with their primary care physician (11). These struggles may be more detrimental for patients who are at a higher-than-average risk of developing diabetes. For example, a national cohort study in Korea found that a diagnosis of cancer increased the risk of subsequent diabetes (12). A case-cohort study in Israel that investigated the association between hormone therapy and diabetes risk among 2,246 female breast cancer survivors found that 48% of diabetes incidence could have been prevented had patients not received hormone therapy (13). Early implementation of a diabetes prevention strategy, particularly for patients with cancer and prediabetes, elevated blood glucose, or active engagement with a primary care physician during cancer treatment, could prevent comorbidity and improve survivorship. Furthermore, cancer treatments such as chemotherapy, radiation, or immunotherapy are associated with a higher prevalence of prediabetes (14). 

Comorbidities or complications associated with cancer are linked to increased health care costs and various kinds of health care use, including ambulatory care visits and emergency department visits (15,16). However, evidence that focuses on the effects of specific kinds of comorbidity, such as diabetes, on health care use is limited. One study that used data from a statewide electronic health record database from 2007 to 2017 in the US found a significant association of having both diabetes and colorectal cancer with emergency department visits but did not examine other outcomes, such as hospitalization, which is a major driver of health care costs (17). Furthermore, little is known about how patterns of health care use differ across stages of diabetes. Addressing these gaps may help to improve the delivery of effective clinical care and preventive services for people with cancer and diabetes.

The objective of this study was to examine the association of health care use patterns among patients with cancer, stratified by diagnosis of diabetes or prediabetes. Findings from the current study may guide research to develop an optimal coordinated care model for early detection of prediabetes or diabetes and to enhance cancer survivorship for people with cancer and comorbidities.

## Methods

Our study used a cross-sectional design and data from the National Health and Nutrition Examination Survey (NHANES) for the 3-year cycle of 2017–2020, before the pandemic. NHANES has been conducted since 1960 and is designed to assess the health and nutritional status of adults and children in the US. It collects nationally representative data through clinical examinations, selected medical and laboratory tests, and self-reported data. NHANES uses a stratified, multistage probability sample design and recommends using weights, stratification, and cluster variables to account for the complex sample design (18). Thus, we applied these variables to the statistical analyses to generate population estimates.

### Study population

Our study population comprised adults aged 18 years or older who were diagnosed with any cancer and had physician-diagnosed diabetes or prediabetes. Those with a cancer history were identified by using the question, “Have you ever been told by a doctor or other health professional that you had cancer or a malignancy of any kind?” Physician-diagnosed diabetes and prediabetes were identified through self-report on the NHANES questionnaire. In addition, to reduce the risk of recall bias, we used NHANES laboratory results of the hemoglobin A_1c_ (HbA_1c_) test. We excluded data on undiagnosed diabetes because the sample size was too small for generating population estimates. We classified people into 3 categories: 1) those with a cancer history only, 2) those with any cancer history and prediabetes, and 3) those with any cancer history and diabetes. We excluded records that had missing data for these variables.

### Outcomes

A primary outcome was the number of visits to a physician’s office, a clinic, or “some other place” in the previous 12 months. This visit did not include hospitalizations, emergency department visits, home visits, or telephone calls. A secondary outcome was an overnight stay in a hospital in the previous 12 months. It excluded overnight stays in the emergency department.

### Independent variable

A primary independent variable was comorbidity status. We categorized the study population into 3 groups: 1) cancer only, 2) cancer and prediabetes, and 3) cancer and diabetes. Control variables were demographic characteristics (age, sex, and race and ethnicity), education, body mass index (BMI), and having a usual source of care (yes or no). We treated age as a continuous variable. Sex was a dichotomous variable (male or female). We categorized race and ethnicity into 4 categories: 1) Hispanic or Latino, 2) non-Hispanic Black, 3) non-Hispanic White, and 4) Other (American Indian or Alaska Native, Asian, and Native Hawaiian or Pacific Islander) or multiracial. We converted education into a dichotomous variable (less than high school and high school graduate or above). Financial status was measured by the ratio of income to poverty (total family income divided by the poverty threshold) and dichotomized into 2 levels: 1) poor (ratio <1) and 2) rich (ratio ≥1). Health status was measured by self-reported general health condition and grouped into 2 levels: 1) fair or above (excellent, very good, good, or fair) and 2) poor. BMI was categorized into 3 levels: 1) normal (BMI, 18.5–24.9), 2) overweight (25.0–29.9), and 3) obese (≥30.0). Health insurance status was categorized into 2 levels: 1) yes, insured, and 2) no, uninsured. We excluded underweight people due to a high risk of mortality and little relevance to our study. Lastly, we treated usual source of care as a dichotomous variable (has a usual source or does not have a usual source of care). We counted the number of other chronic diseases reported by the survey respondent, such as arthritis, cancer (if the respondent has ≥1 cancers), cardiovascular diseases (eg, congestive heart failure, coronary heart disease, angina, or stroke), chronic kidney disease, depression, hypertension, and pulmonary diseases (eg, emphysema, chronic bronchitis, or asthma). We categorized these data into 4 groups: 1) no other comorbidity, 2) 1 additional comorbidity, 3) 2 additional comorbidities, and 4) ≥3 additional comorbidities.

### Statistical analysis

We conducted a descriptive analysis of the baseline characteristics of the 3 groups of NHANES respondents (cancer only, cancer and prediabetes, and cancer and diabetes). We used χ^2^ tests and *t* tests to determine significant differences between groups, with *P* < .05 considered significant. We used a Poisson regression model to determine the effect of comorbidity status (cancer only, cancer and prediabetes, and cancer and diabetes) on the number of health care visits in the previous 12 months. We used a multivariate logistic regression model to examine the risk of an overnight hospital stay associated with comorbidity status. We conducted both unadjusted and adjusted models. The Poisson regression model produced incident rate ratios (IRRs) and 95% CIs, and the multivariate logistic regression model produced odds ratios (ORs) and 95% CIs. The Pearson χ^2^ test was used to evaluate the goodness-of-fit for the Poisson regression model, and the Akaike Information Criterion (AIC) was used to evaluate the goodness-of-fit for the multivariate logistic regression model. We used SAS version 9.4 (SAS Institute, Inc) for all analyses. This study was exempted from the University of Florida Institutional Review Board review because of the use of publicly available data. We followed the STROBE statement in conducting methods and reporting results (19).

## Results

The unweighted sample size was 905, representing 27,180,715 people in the US. Of these cancer patients, 24.4% (weighted percentage) had a type 2 diabetes diagnosis, and 25.8% (weighted percentage) had a prediabetes diagnosis (Table 1). The mean age of the total study population was 63.9 years. People with cancer and diabetes (mean age, 68.8 y) and people with cancer and prediabetes (mean age, 66.7 y) were older, on average, than people with cancer only (mean, 59.9 y). The percentage of people with less than a high school diploma was significantly larger among people with cancer and diabetes (10.2%) and cancer and prediabetes (9.4%) than people with cancer only (5.2%). The percentage of people who had a BMI in the obese range was significantly larger among people with cancer and diabetes (63.3%) and cancer and prediabetes (43.9%) than people with cancer only (30.7%). The percentage of people with 3 or more additional comorbidities was significantly larger among people with cancer and diabetes (51.0%) and cancer and prediabetes (30.3%) than among people with cancer only (17.1%). Regardless of comorbidity status, more than 95% of people had health insurance. The percentage of people with a usual source of care was significantly larger among people with cancer and diabetes (98.3%) and cancer and prediabetes (97.2%) than among people with cancer only (91.6%).

**Table 1:** Baseline Characteristics of Adults With Cancer, Stratified by Diabetes Status, National Health and Nutrition Examination Survey, 2017–2020^a^

Characteristic	Cancer only	Cancer and prediabetes	Cancer and diabetes	*P* value^b^
**Unweighted sample size**	403	248	254	—
**Weighted sample size, no. (%)**	13,532,512 (49.8)	7,024,691 (25.8)	6,623,512 (24.4)	—
**Mean age, y**	59.9	66.7	68.8	<.001
**Sex**				
Male	40.8	38.1	46.4	.51
Female	59.2	61.9	53.6	
**Race and ethnicity**				
Hispanic	6.0	19.4	8.6	.33
Non-Hispanic Black	5.8	32.1	6.3	
Non-Hispanic White	82.2	26.3	79.3	
Other^c^	6.1	19.3	5.8	
**Education**				
Less than high school	5.2	9.4	10.2	.02
High school graduate or above	94.8	90.6	89.8	
**Financial status^d^**				
Poor	6.3	5.7	8.9	.33
Rich	93.7	94.3	91.1	
**Body mass index, calculated as weight (kg) divided by height in meters squared**				
Normal (18.5–24.9)	32.5	16.0	6.5	<.001
Overweight (25.0–29.9)	36.8	40.1	30.2	
Obese (≥30.0)	30.7	43.9	63.3	
**Health status**				
Fair or above	95.7	95.2	89.1	.02
Poor	4.3	4.8	10.9	
**No. of additional comorbidities**				
0	30.9	19.2	5.6	<.001
1	30.8	21.1	17.6	
2	21.3	29.4	25.8	
≥3	17.1	30.3	51.0	
**Has a usual source of care**				
No	8.4	2.81	1.8	.02
Yes	91.6	97.2	98.2	
**Health insurance**				
No	3.5	1.4	4.8	.25
Yes	96.5	98.6	95.2	
**No. of health care visits in previous 12 months^e^**	3.4	3.8	3.8	.13
**Had an overnight stay in a hospital in previous 12 months^f^**				
No	15.3	15.5	31.6	<.001
Yes	84.7	84.5	68.4	

a All values are weighted percentages, unless otherwise indicated.

b Determined by *t* test for continuous variable and χ^2^ tests for categorical variables.

c Includes American Indian or Alaska Native, Asian, and Native Hawaiian or Pacific Islander, and multiracial.

d Measured by the ratio of income to poverty (total family income divided by the poverty threshold) and dichotomized into 2 levels: 1) poor (ratio < 1) and 2) rich (ratio ≥ 1).

e Visits to a physician’s office, a clinic, or some other place in the previous 12 months, not including hospitalizations, emergency department visits, home visits, or telephone calls.

f Excludes overnight stays in the emergency department.

In the unadjusted Poisson regression model, the IRR for the number of health care visits in the previous 12 months was significantly higher among people with cancer and diabetes (IRR = 1.19; 95% CI, 1.12–1.27; *P* < .001) than among people with cancer only (Table 2). However, after controlling for covariates, the comorbidity of cancer and diabetes was not significantly associated with increases in the number of health care visits (IRR = 1.04; 95% CI, 0.94–1.15; *P* = .44). After controlling for covariates, the comorbidity of cancer and prediabetes was associated with increases in the number of health care visits in the previous 12 months (IRR = 1.11; 95% CI, 1.01–1.22; *P* = .03). The results of the goodness-of-fit test for both unadjusted and adjusted models were not significant, indicating that neither model fit the data well.

**Table 2:** Results of Poisson Regression for the Number of Health Care Visits^a^ in the Previous 12 Months, National Health and Nutrition Examination Survey, 2017–2020

Characteristic	Unadjusted IRR (95% CI) [*P* value]	Adjusted IRR (95% CI)^b^ [*P* value]
Cancer only	Reference	Reference
Cancer and prediabetes	1.05 (0.98–1.12) [.14]	1.11 (1.01–1.22) [.03]
Cancer and diabetes	1.19 (1.12–1.27) [<.001]	1.04 (0.94–1.15) [.44]

Abbreviation: IRR, incidence rate ratio.

a Visits to a physician’s office, a clinic, or some other place in the previous 12 months, not including hospitalizations, emergency department visits, home visits, or telephone calls.

b Controlled for age, sex, race and ethnicity, education, poverty-to-income ratio, body mass index, number of additional comorbidities, and health insurance.

In the multivariate logistic regression, the unadjusted model showed that people with diabetes and cancer were 2.5 times more likely than people with cancer only to stay overnight in a hospital (OR = 2.55; 95% CI, 1.54–4.21). However, after controlling for covariates, this association was not significant (OR = 1.57; 95% CI, 0.82–3.02). Moreover, we found no significant association in comorbidity with prediabetes for the risk of an overnight stay in a hospital in either the unadjusted or adjusted model (Table 3). The goodness-of-fit test for the adjusted model had a lower AIC value than the unadjusted model, indicating a better fitting model.

**Table 3:** Results of Multivariate Logistic Regression for Risk of Overnight Stay in a Hospital^a^ in the Previous Year, National Health and Nutrition Examination Survey, 2017–2020

Characteristic	Unadjusted odds ratio (95% CI)^b^	Adjusted^c^ odds ratio (95% CI)^b^
Cancer only	1 [Reference]	1 [Reference]
Cancer and prediabetes	1.01 (0.63–1.64)	0.84 (0.42–1.65)
Cancer and diabetes	2.55 (1.54–4.21)	1.57 (0.82–3.02)

a Excludes overnight stays in the emergency department.

b An odds ratio with a 95% CI that includes 1 indicates no significant effect on risk.

c Controlled for age, sex, race and ethnicity, education, poverty-to-income ratio, body mass index, number of comorbidities, and health insurance.

## Discussion

The objective of our study was to examine patterns of health care use among people with cancer and either prediabetes or diabetes. In our nationally representative sample, patients with cancer and diabetes had 19% more health care visits than people with cancer only according to the unadjusted regression model, and patients with cancer and prediabetes had 11% more health care visits than people with cancer only according to the adjusted regression model. Future studies may be needed to test strategies to improve care coordination and early initiation of preventive care strategies for people with cancer at risk of developing prediabetes and diabetes.

Having diabetes and cancer increased the risk for an overnight stay in a hospital in the unadjusted regression models, whereas having prediabetes and cancer increased the number of health care visits in the adjusted regression model only. These findings indicate that different stages of diabetes may drive different health care needs. In the qualitative study conducted in 2021 and 2022 at 3 New York City hospitals among 15 women with breast cancer and either diabetes or prediabetes, 7 participants reported glucose levels of more than 200 mg/dL (normal is 70–90 mg/dL) and 9 participants indicated a lack of glucose control during cancer treatment (11). In addition, as cancer treatment tends to be prioritized over other treatment, diabetes prevention and management led by a primary care physician may be paused (20). Medication adherence for chronic diseases may also decline due to the priority of cancer treatment (21,22). In addition, many cancer patients with comorbidities may not receive self-management education or guidelines for preventive care, negatively affecting cancer survivorship (23). Moreover, our study found that patients with cancer and diabetes were 2 times more likely to be hospitalized, whereas patients with cancer and prediabetes did not have significantly higher rates of hospitalization. This finding was supported by literature showing that patients with cancer and at least 1 comorbidity were more likely than patients with no comorbidities to be hospitalized (6,24). Clinical guidelines for managing patients with cancer and prediabetes are lacking, and communication guidelines for coordinated care between oncologists and primary care physicians are limited. Because many patients with cancer tend to prioritize cancer treatment over primary care for prediabetes or diabetes, detrimental clinical outcomes and increased health care use may not be preventable without early prevention or ongoing management. In response to increases in the prevalence of prediabetes and cancer, it is important to develop a systematic preventive care model for early-stage chronic diseases (eg, prediabetes, prehypertension) that includes collaboration between oncologists and primary care physicians. Such a model could be a cost-effective strategy for improving cancer survivorship.

Our study also found that more than 80% of comorbid people were overweight or obese (compared with 67.5% among those with cancer only). It is well established that obesity is significantly associated with cancer incidence and mortality (25) and is a risk factor for cancer and chronic diseases (eg, diabetes, prediabetes) (26,27). Excessive body fat causes chronic inflammation that may be attributed to cancer treatment–associated adverse outcomes (25). Thus, it is important to control overweight and obesity during cancer treatment. A combination of diet and exercise was identified as a more effective intervention for weight loss than a standard of care for patients with cancer (28). Clinicians need to provide self-management guidelines for lifestyle changes when a cancer diagnosis is first made, especially among overweight or obese patients. In the qualitative study among 15 women with breast cancer and either diabetes or prediabetes, participants indicated not receiving guidance on self-management or having a designated clinician who continuously monitored them (11). One in-depth patient interview found that a patient searched for diet or exercise information on Google (11). This research suggests a need for self-management guidelines provided by clinicians for controlling overweight or obesity and monitoring chronic disease progression.

Educational attainment was significantly associated with comorbidity status. Among patients with less than a high school diploma, the percentage of patients with a comorbidity was twice the percentage of patients with no comorbidity (9.4% and 10.2% vs 5.2%). Education may be key to health behaviors and the prevention of adverse outcomes. It is well established that education inequality is associated with cancer survivorship (29,30). For example, a study in The Netherlands showed that among patients with cancer and comorbidity, those with a low level of education (equivalent to primary school) had a 3 times higher risk of death than those with a university degree (29). A study of education differentials in cancer deaths in Lithuania found an inverse educational gradient for selected cancer sites among men and women, noting that substantial shares of cancer deaths (8% to 35%) could have been avoided or postponed (30). Increasing access to resources for patients with low levels of education may help to minimize the number of comorbidities that can arise and ultimately improve their cancer survivorship. Particularly, providing more resources may benefit from developing effective and structured communication strategies with providers.

Optimal coordinated care is crucial to mitigate the burden of comorbidities on health care use and costs among patients with cancer. Despite the growing need for increased care coordination between primary care physicians and oncologists, no standardized care coordination model exists for managing the comorbidity of cancer and chronic diseases such as prediabetes or diabetes (31). Additionally, the involvement of primary care physicians in cancer care is limited, especially during active cancer treatment (32). Previous research identified some barriers to effective cancer care coordination, including inadequate communication between oncologists and primary care providers and between patients and primary care providers; geographic limitations; and limited interoperability of the electronic health record among health care providers (32,33). Fortunately, the recent rapid technological evolution has provided new opportunities to reduce these barriers. Studies conducted at the Johns Hopkins Primary Care for Cancer Survivors clinic in 2015 and the Duke Cancer Institute during 2020–2021 found that comorbid patients were more likely to use telehealth for cancer and primary care, and telehealth improved outcomes such as patient satisfaction and survivorship (34,35). Using artificial intelligence in the care coordination process and communication will become pivotal to improving an efficient and effective care coordination model. An optimal care coordination model integrated with technology can be achieved by using standardized communication channels among health care providers and between health care providers and patients and the interoperability of electronic health records. Moreover, appropriate data privacy and security regulation will be essential to ensure patient trust in the care coordination model. To leverage these benefits, standardized clinical guidelines for managing comorbidities in patients with cancer should be developed. These guidelines would provide clear recommendations on integrating care coordination.

### Limitations

Our study has several limitations. First, the diagnosis information obtained from a self-reported survey may be subject to recall bias, and we could not determine the exact timing of the diagnosis of diabetes or prediabetes and cancer. Second, our study used cross-sectional data, which prevented us from following disease progression over time and examining the effects of various treatments. A study that uses longitudinal data is needed to understand the effect of comorbidity on health care use among cancer patients. Third, we could not identify the reasons for health care use because of a lack of data. A study that incorporates electronic health records may identify patient-centered health care needs for those with comorbidities. Lastly, while the study identified patients with cancer who had undiagnosed diabetes, the sample size was too small to generate population estimates. Studies that use larger data sets could examine the role of undiagnosed diabetes on cancer prognosis and outcomes.

### Conclusion

Among people with cancer, diabetes was significantly associated with an increased risk of an overnight hospital stay, whereas prediabetes was significantly associated with an increase in the number of health care visits. Our findings suggest that it may be beneficial to prioritize preventive measures (eg, screening) to prevent prediabetes from progressing to diabetes in patients with cancer and develop optimal coordinated care, which could help alleviate the strain on the health care system and improve oncology care.
